# Severe Conduction Disturbances Accompanying Anterior Wall Myocardial Infarction: An Infrequent Presentation to Remember

**DOI:** 10.1155/cric/8331292

**Published:** 2025-05-24

**Authors:** Manuel Mallol-Simmonds, Rocio Fuentes-Garrido, Alfredo Villarroel, Catalina Valenzuela, Marcelo Llancaqueo

**Affiliations:** ^1^Cardiovascular Investigation Unit, University of Chile's Clinical Hospital, Santiago, Chile; ^2^Faculty of Medicine, University of Chile, Santiago, Chile

**Keywords:** anterior wall myocardial infarction, atrioventricular block, bundle branch block, cardiogenic shock

## Abstract

**Background:** Anterior ST-elevation myocardial infarction (STEMI) is frequently associated with severe complications, such as myocardial dysfunction and conduction abnormalities. Complete atrioventricular block (CAVB), especially when combined with bifascicular block, is a rare but critical presentation that reflects extensive myocardial damage, often linked to proximal left anterior descending (LAD) artery occlusion.

**Case Presentation:** A 76-year-old male with a history of hypertension, diabetes mellitus, and dyslipidemia presented to the emergency department with a 6-day history of gastrointestinal symptoms and recurrent syncope. On arrival, he exhibited bradycardia, hypotension, and mottling, progressing to cardiac arrest. The return of spontaneous circulation was achieved after one cycle of advanced cardiovascular life support. Electrocardiography revealed CAVB with a ventricular escape rhythm displaying right bundle branch block morphology, left anterior fascicular block, and anterior ST-segment elevation. Emergency interventions included endotracheal intubation, Swan–Ganz catheterization, transvenous pacing, and vasopressor support. Echocardiography demonstrated severe left ventricular systolic dysfunction with an ejection fraction of 25%. Coronary angiography identified critical proximal stenoses in the LAD and circumflex arteries, managed with percutaneous coronary intervention and stent placement. Persistent conduction abnormalities and systolic dysfunction necessitated implantation of a cardiac resynchronization therapy defibrillator (CRT-D). Despite extensive rehabilitation efforts, the patient died after 60 days of hospitalization.

**Discussion:** This case highlights the importance of rapid recognition and intervention in anterior STEMI complicated by CAVB and bifascicular block, signaling significant proximal LAD involvement. Advanced therapeutic options, including CRT-D, are crucial for addressing these rare, life-threatening conduction disturbances. The fatal outcome underscores the need for vigilant follow-up and individualized preventive strategies to improve prognosis in such complex STEMI cases.

## 1. Introduction

Cardiovascular disease remains a leading global cause of mortality. According to the most recent report from the World Health Organization (WHO) and the Global Burden of Disease (GBD) study from 2019, cardiovascular diseases caused approximately 18.6 million deaths, representing nearly 32% of all global deaths that year. This corresponds to an age-standardized global rate of 233 deaths per 100,000 people, with significant variations across regions.

Data from the Pan American Health Organization (PAHO) report 137 deaths per 100,000 inhabitants due to cardiovascular diseases in the year 2019, presenting a persistent public health challenge [1]. Among acute coronary syndromes, ST-elevation myocardial infarction (STEMI) is notable for its high early morbidity and mortality, with outcomes heavily reliant on timely reperfusion therapy to restore coronary blood flow. STEMI-related complications can be categorized by mechanism—electrical (e.g., arrhythmias), mechanical (e.g., ventricular dysfunction), thrombotic (e.g., stent thrombosis), or hemorrhagic (e.g., bleeding from anticoagulation)—or by timing, occurring as early or late events. In anterior STEMI, typically resulting from left anterior descending (LAD) artery occlusion, myocardial dysfunction is the most common complication, often progressing to heart failure or cardiogenic shock due to extensive myocardial necrosis [2]. Although less frequent, conduction abnormalities carry significant prognostic weight. Complete atrioventricular block (CAVB), for example, is independently associated with increased mortality and major adverse cardiovascular events (MACEs) [3, 4]. Moreover, CAVB is linked to higher rehospitalization rates and more severe systolic dysfunction [5], even in patients with successful reperfusion, underscoring the persistent electrical and hemodynamic burden despite optimal revascularization.

## 2. Case Presentation

**Figure 1:**
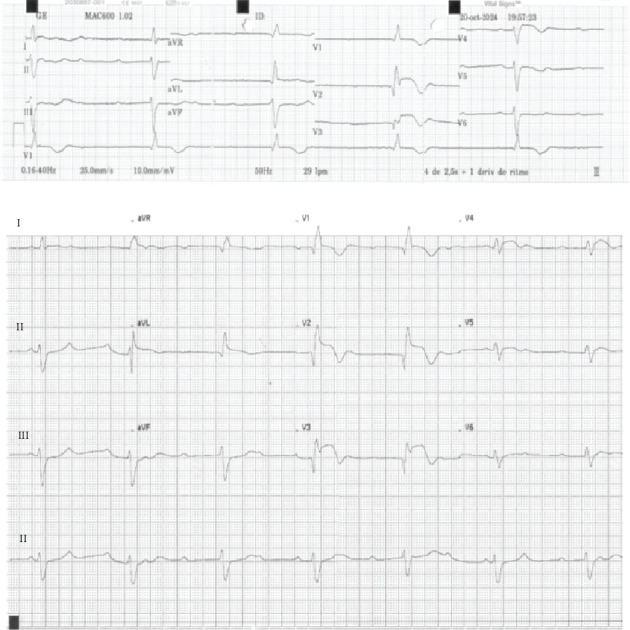
Twelve-lead ECG in the emergency department. (a) Initial ECG showing CAVB, ventricular escape rhythm with RBBB morphology at 32 bpm, LAFB, ST-segment elevation from V1 to V4. (b) Follow-up ECG at 30 min, demonstrating increased ST-segment elevation and Q waves from V1 to V3, mild ST-segment elevation in aVL, and persistence of electrical disturbances described above.

A 76-year-old male with a history of hypertension, Type 2 diabetes mellitus, and dyslipidemia presented with a 6-day prodrome of vomiting, diarrhea, and progressive dizziness, followed by two syncopal episodes within 48 h, the second associated with minor cranial trauma. On arrival at the emergency department, he exhibited profound bradycardia (32 beats per minute (bpm)), hypotension, peripheral hypoperfusion, and an acute confusional state. Within minutes, he experienced cardiac arrest, with return of spontaneous circulation achieved after one cycle of advanced cardiovascular life support. Initial 12-lead electrocardiography (ECG) revealed CAVB with a ventricular escape rhythm at 32 bpm, displaying right bundle branch block (RBBB) morphology, left anterior fascicular block (LAFB), and ST-segment elevation from V2 to V4, rapidly extending to V6 (Figure 1). Two transient Stokes–Adams attacks were observed. Emergent stabilization included orotracheal intubation, temporary transvenous pacemaker placement achieving ventricular capture at 70 bpm, and insertion of a Swan–Ganz catheter (SGC). Initial SGC-derived hemodynamic parameters showed a cardiac output of 2.1 L/min, cardiac index of 1.1 L/min/m^2^, systemic vascular resistance index of 2235 dyn·s/cm^5^, pulmonary capillary wedge pressure of 17 mmHg, cardiac power output of 0.58 W, and pulmonary artery pressures of 36/22/27 mmHg (systolic/diastolic/mean). Pharmacologic support with norepinephrine and dobutamine was initiated, guided by SGC findings, while the catheterization laboratory was activated.

**Figure 2:**
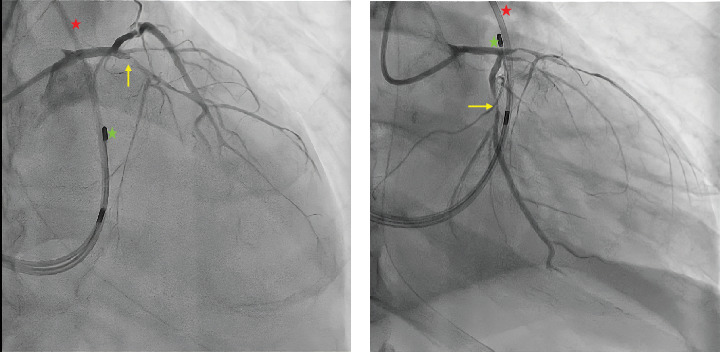
Left coronary angiography. (a) Right anterior oblique 20°–cranial 30° projection showing a severe focal lesion in the proximal LCx (yellow arrow). (b) Right anterior oblique 20°–caudal 20° projection showing a diffusely diseased LAD with a severe proximal lesion (yellow arrow). Both images incidentally display the SGC (red star) and temporary transvenous pacemaker (green star).

Coronary angiography revealed diffuse atherosclerosis, including severe proximal stenosis in the LAD with TIMI 2 flow, a moderately developed circumflex artery (LCx) with 90% proximal obstruction (TIMI 2 flow), and a right dominant coronary artery (RCA) with no significant stenosis (Figure 2). Percutaneous coronary intervention (PCI) was performed, using a paclitaxel-coated balloon for the proximal LAD lesion and a drug-eluting stent (Onyx 3.0/18 mm) in the proximal LCx, both arteries achieving TIMI 3 flow.

CAVB resolved immediately post-PCI, though LAFB and RBBB persisted. Echocardiography revealed severe systolic dysfunction, with an ejection fraction (LVEF) of 25% with nonthinned akinesia of the midapical anterior, lateral, septal, anteroseptal, and inferolateral walls. At 36 h postadmission, the patient developed atrial fibrillation, followed by intermittent CAVB and a severe concomitant low cardiac output state. Persistent systolic dysfunction was confirmed 10 days postadmission, prompting implantation of a cardiac resynchronization therapy with defibrillator (CRT-D). Subsequent clinical improvement allowed initiation of guideline-directed medical therapy for heart failure with reduced LVEF (HFrEF), titrated to macrohemodynamic tolerance. However, due to the initial and life-threatening critical illness, he developed severe ICU-acquired myopathy, dysphagia, neuropathy, and sarcopenia, requiring endoscopic gastrostomy tube placement and intensive rehabilitation. Three weeks later, he experienced septic shock secondary to healthcare-associated pneumonia, progressing rapidly to multiple organ failure and death on Day 60 of hospitalization.

## 3. Discussion

Conduction disturbances in anterior STEMI vary widely, primarily depending on the culprit vessel. The right bundle branch of the His–Purkinje system is predominantly supplied by the septal branch of the LAD, either alone or with the atrioventricular (AV) nodal artery, while the left anterior fascicle relies solely on LAD perfusion. The His bundle benefits from dual blood supply via the AV nodal artery and the first septal perforator of the LAD. Coexisting RBBB and LAFB in anterior STEMI strongly suggest proximal LAD occlusion [2]. CAVB occurs in approximately 1.7% of anterior STEMI cases and is associated with significantly higher rates of all-cause mortality and MACE (82%) compared to anterior STEMI without CAVB (20% [6]). In contrast, CAVB is more common in inferior STEMI, with its etiology varying by vascular territory. Culprit lesions in the RCA confer an odds ratio of up to 3.9 for CAVB [7], a risk comparable to that in Mobitz II second-degree AV block and high-grade AV block [8]. Nevertheless, the angiography in this case did not reveal coronary occlusion at the time of presentation. It is possible that a prior occlusion caused ischemic damage to the AV node, with the artery having spontaneously reperfused. Another possibility is the presence of pre-existing damage (e.g., fibrosis) in the AV node, which, in the context of significant ischemia not mediated by coronary occlusion, could have led to CAVB. The above can only explain potential etiological options for the CAVB. However, the coexistence of bifascicular block and CAVB, as well as the subsequent changes described, is, with high probability, secondary to the extension of the myocardial infarction.

The prognosis of conduction disturbances in STEMI is closely tied to the infarcted wall. In inferior STEMI, CAVB typically progresses from first-degree AV block to a supra-Hisian block with a narrow QRS complex, caused not only by ischemic mechanisms but also by vagotonic pathways such as the Bezold–Jarisch reflex [9]. In anterior wall infarction, however, CAVB often presents with an infranodal escape rhythm, leading to severe hemodynamic instability, pronounced systolic dysfunction, and a frequent need for permanent pacing or advanced device therapy [10, 11].

Despite comprehensive management in both the acute and late phases, the outcome in this case could not be altered. The atypical, delayed presentation—likely with gastrointestinal symptoms as an anginal equivalent [12]—and syncope in the described context were probable key contributors to the adverse evolution.

This case illustrates a rare and severe presentation of anterior STEMI manifesting as an electrical disturbance, resulting in cardiogenic shock and severe systolic dysfunction, ultimately requiring CRT-D implantation among other interventions. It highlights that, despite advanced therapeutic efforts, this clinical scenario carries substantial mortality, emphasizing the need for early consultation and aggressive interventions to potentially modify its natural course.

Informed consent for scientific publication was provided by the patient's immediate family.

## 4. Take Away

“*Time is heart*.” The interval between first medical contact and guidewire crossing or fibrinolytic administration is manageable by healthcare systems; however, ischemic time—which encompasses the duration prior to medical contact—also depends on the patient's time to seek care. Together, these factors critically influence prognosis. CAVD, LAFB, and RBBB may arise secondary to anterior STEMI, constituting a rare presentation with distinct challenges and typically unfavorable outcomes.

## Data Availability

In accordance with Chilean legislation, including Law No. 19.628 on the Protection of Private Life and the regulations governing research involving human subjects (as outlined in the Guidelines for Ethical Conduct in Health Research of the Chilean Ministry of Health), all personal and clinical information that could directly or indirectly identify the patient is strictly protected and cannot be disclosed. As such, no additional data beyond those figures and clinical details explicitly authorized for publication in this manuscript are available. All data presented have been anonymized and included with the informed consent of the patient, in full compliance with national ethical and legal standards for biomedical research.
